# Role of macrophage-to-myofibroblast transition in chronic liver injury and liver fibrosis

**DOI:** 10.1186/s40001-023-01488-7

**Published:** 2023-11-08

**Authors:** Suhong Xia, Yujie Huang, Yu Zhang, Mingyu Zhang, Kai Zhao, Ping Han, Dean Tian, Jiazhi Liao, Jingmei Liu

**Affiliations:** Department of Gastroenterology, Tongji Hospital of Tongji Medical College, Huazhong University of Science and Technology, Wuhan, 430030 Hubei Province China

**Keywords:** Macrophage, Macrophage to myofibroblast transition, Chronic liver injury, Liver fibrosis

## Abstract

**Background:**

Chronic liver injury contributes to liver fibrosis, which is characterized by the excessive deposition of extracellular matrix (ECM) components. ECM is mainly composed of myofibroblasts. Recently, macrophage-to-myofibroblasts transition (MMT), has been identified as a novel origin for myofibroblasts. However, the potential functions of MMT in chronic liver injury and liver fibrosis remain unknown.

**Methods:**

To clarify the transformation of fibrotic cells in hepatic fibrosis**,** liver specimens were collected from people at different stages in the progression of hepatic fibrosis and stained with immunofluorescence. Models of hepatic fibrosis such as the CCL4 model, HFD-induced NAFLD model, MCD-induced NAFLD model and ethanol-induced AFLD model were demonstrated and were stained with immunofluorescence.

**Results:**

Here, we uncovered macrophages underwent MMT in clinical liver fibrosis tissue samples and multiple animal models of chronic liver injury. MMT cells were found in specimens from patients with liver fibrosis on the basis of co-expression of macrophage (CD68) and myofibroblast (a-SMA) markers. Moreover, macrophages could transform into myofibroblasts in CCL4-induced liver fibrosis model, high-fat diet (HFD) and methionine-choline-deficient diet (MCD)-induced nonalcoholic fatty liver diseases (NAFLD) model, and ethanol-induced alcoholic fatty liver diseases (AFLD) model. In addition, we highlighted that MMT cells mainly had a predominant M2 phenotype in both human and experimental chronic liver injury.

**Conclusions:**

Taken together, MMT acts a crucial role in chronic liver injury and liver fibrosis.

## Background

Liver fibrosis exhibited pathophysiological reactions to chronic liver injury, such as viral infection, drug toxicity, alcoholic and non-alcoholic fatty liver diseases [1]. The excessive accumulation of aberrant extracellular matrix (ECM) components in the liver, particularly the collagen I and α-SMA, were defined as the characteristic feature of liver fibrosis [2]. The hepatic stellate cells (HSCs) differentiated into myofibroblasts (MFs), which lead to the deposition of ECM and contributed to the desmoplastic of the liver [3]. Although many potential anti-fibrotic targets have been identified, effective treatment to prevent or reverse liver fibrosis doesn’t exist yet.

Myofibroblasts, the primary source of collagen Type I for fibrous scarring, are absent in the healthy liver [4]. During liver fibrosis, activated hepatic stellate cells (HSCs) are the major source of myofibroblasts [5]. In addition, myofibroblasts can also be derived from epithelia cells and endothelia cells through epithelial-to-mesenchymal transition (EMT) and endothelial-to-mesenchymal transition (EndoMT), respectively [6, 7]. Recently, researches indicate that monocytes/macrophages can differentiated to myofibroblast by macrophage-to-myofibroblast transition (MMT) [8, 9].

Accumulating evidence indicates that MMT plays a crucial role in the development of fibrotic disorders, including kidney fibrosis, lung fibrosis and subretinal fibrosis [8, 10, 11]. MMT is the transformation of macrophages into myofibroblasts in response to an inflammatory stimulation, which can produce collagen. The characteristic of MMT cells is the co-expression of macrophage markers (CD68 or F4/80) and myofibroblast marker α-smooth muscle actin (α-SMA) [8]. Studies have shown that macrophages played an important role in the process of liver fibrosis [12]. However, the role of MMT in liver fibrosis is still not understood.

In this study, we uncovered macrophages underwent MMT in clinical liver fibrosis patient samples and CCl4-induced liver fibrosis model. Moreover, MMT is also present in high-fat diet (HFD) and methionine-choline-deficient diet (MCD)-induced non-alcoholic fatty liver diseases (NAFLD) model, and ethanol-induced alcoholic fatty liver diseases (AFLD) model. Furthermore, the majority of MMT cells in human and experimental chronic liver injury samples was the M2 macrophages. Our findings indicated that MMT may be an underlying mechanism of liver fibrosis caused by different causes of liver injury.

## Methods

### Patient samples

Totally, the clinical liver tissue samples were acquired from surgical resection without preoperative treatment at Tongji Hospital of Tongji Medical College, Huazhong University of Science and Technology (HUST) (Wuhan, China). Hematoxylin and eosin (H&E) from the paraffin-embedded 5 μm thick slides were performed on the samples. Liver tissues were examined by pathologists experienced in liver diseases. Then, the liver tissues were classified according to the degree of fibrosis, S represents the stage of liver fibrosis. Written informed consent forms were provided and communicated to all patients. This study was approved by the Ethics Committee of Tongji Hospital, and it is compliant with the guidelines of the Declaration of Helsinki.

### Animals and animal models

All animal procedures were performed following the Guide for the Care and Use of Laboratory Animals and standards articulated in the Animal Research: Reporting of In Vivo Experiments. All animal experiments were approved by the Committee on the Tongji Hospital of Tongji Medical College, Huazhong University of Science and Technology. The C57BL/6 mice (male, 6 weeks old) were housed and cared for according to the institutional guidelines for animal care.

For CCl4-induced liver fibrosis model (*n* = 8 each group), 25% CCl4 was dissolved using olive oil, and then, the mice were intraperitoneally injected with CCl4 (0.5 ml/kg body weight, twice per week for 4 or 8 weeks) or an equivalent amount of olive oil. Two days after the last injection, the mice were sacrificed, and livers were obtained for further study.

For HFD-induced NAFLD model (*n* = 8 each group), mice were given either a standard chow diet as control or an high-fat diet (HFD) diet with 60% kcal from fat for 16 weeks.

For MCD-induced NAFLD model (*n* = 8 each group), mice were given a methionine/choline-deficient (MCD) diet and normal chow (NC) as control. After 4 weeks of feeding, the mice were sacrificed, and livers were obtained for further study.

For the ethanol-induced AFLD model (*n* = 8 each group), modeling process consists of three phases: liquid feed adaptation period (5 days), modeling period (10 days), and gavage (1 time), which takes 16 days in total. In the first period, all mice were fed the control Lieber-DeCarli diet without restraint. In the second period, the ethanol feeding group was given an ethanol Lieber-DeCarli diet containing 5% alcohol, and the control group was given feeding according to the average intake of the experimental group for 10 days. On day 16, when the gavage was administered mice were treated with a large dose of isocaloric ethanol (5 g/kg body weight). For the control groups, a control liquid diet and then added dextrin in the final stage. After 9 h, the mice were sacrificed, then livers were used for further study.

### Immunofluorescence and image analysis

Immunofluorescence was detected on 5-μm-thick. Baking at 60 °C for an hour, the tissue sections were deparaffinized in xylene and dehydrated by gradient ethanol immersion. Then 3% (vol/vol) hydrogen peroxide was used to block endogenous peroxidase activity. The sections were incubated with mixed primary antibodies overnight in a humid chamber at 4 ℃. Then, multiple fluorescence-labeled secondary antibodies from different species had incubated in sections for 45 min at room temperature. The clinical liver tissues were stained for CD68 (OriGene Technologies, TA802949S, 1:200), CD206 (R&D SYSTEMS, # P22897, 1:200) and α-SMA (Abcam, ab124964, 1:200), DAPI (Promoter, Wuhan, China) was used to stain the nuclei. The mouse liver tissues were stained for F4/80 (ThermoFisher, #14-4801-82, 1:200), CD206 (R&D SYSTEMS, AF2535, 1:200) and α-SMA (Abcam, ab124964, 1:200). Digital images were obtained using a fluorescence microscope (Olympus, Japan). Images were gathered on a fluorescence microscope from single MMT cells co-expressing F4/80 (or CD68), α-SMA and CD206. Five high-power fields were randomly selected from the image, and then double or triple-positive cells were counted as per square millimeter by Image J.

### Statistical analysis

All data are recorded as the mean ± S.D. Statistical analyses using one-way analysis of variance (ANOVA), followed by Tukey's post hoc tests using GraphPad Prism 5, *P* ≤ 0.05 was considered as statistically significant.

## Results

### MMT in the human liver fibrosis sample

**Fig. 1:**
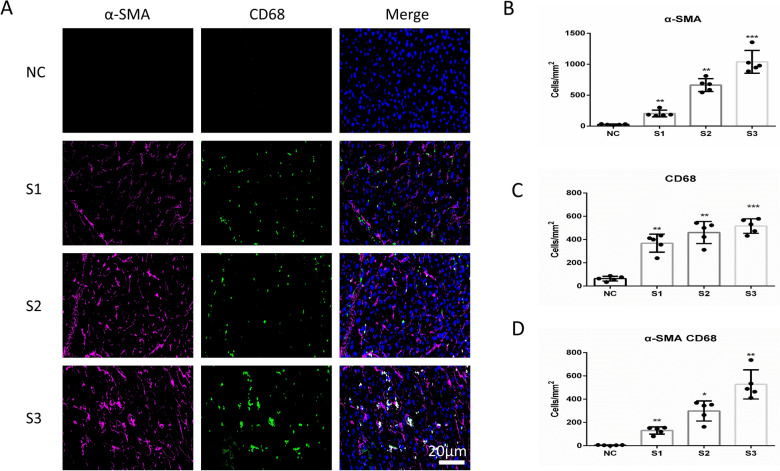
MMT in the human liver fibrosis samples. **A** Representative images of immunofluorescent multi-staining for macrophage marker CD68 (Green) and myofibroblast marker α-SMA (purple) in human liver fibrosis samples. S represented the stage of liver fibrosis. **B** Quantitative data for the expression of α-SMA in human liver fibrosis samples. **C** Quantitative data for the expression of CD68 in human liver fibrosis samples. **D** Quantitative data for the co-expression of CD68 and α-SMA in human liver fibrosis samples. All the data were shown as the mean ± s.d. *P < 0.05, **P < 0.01 and ***P < 0.001 vs. NC group. Scale bar, 20 µm

We sought to authenticate the role of MMT in liver fibrosis. Co-immunostaining of macrophage (CD68) and myofibroblast (α-SMA) was used to denote MMT cells in liver tissue samples, and representative images were demonstrated. As shown in Fig. 1A and B, the expression of a-SMA gradually increased with the aggravation of liver fibrosis. Interestingly, sharply increased expression of macrophage marker CD68 was also found in liver fibrosis tissues (Fig. 1C). Furthermore, we found that co-expression of CD68 and a-SMA was found elevated in liver fibrosis tissues and hardly any in the normal liver tissues (Fig. 1D), which indicated that MMT cells were present in liver fibrosis. In summary, these results indicated that MMT mainly contributed to the progress of liver fibrosis.

### MMT in the CCl4-induced liver fibrosis model

**Fig. 2:**
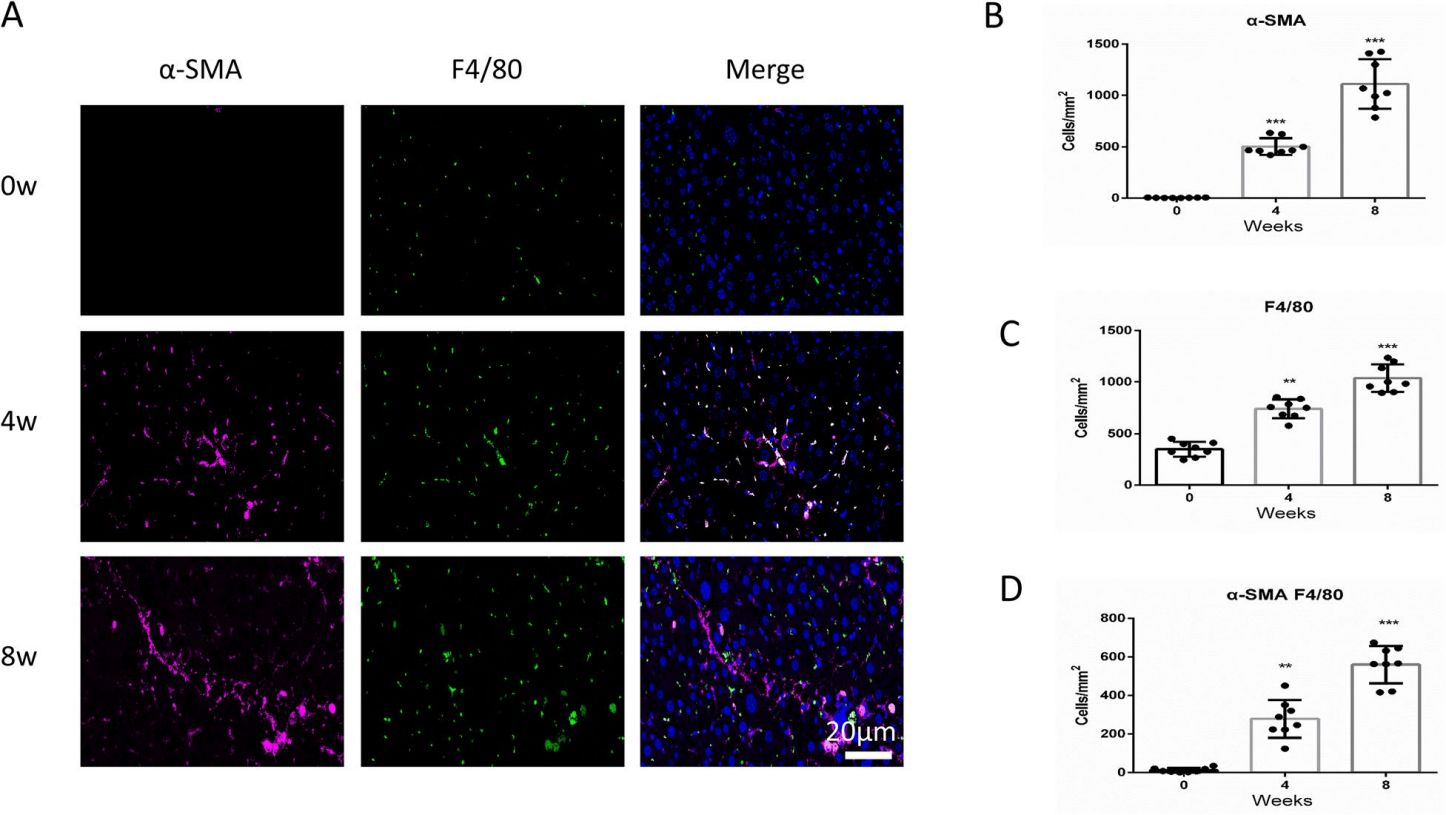
MMT in CCl_4_-induced liver fibrosis model. **A** Representative images of immunofluorescent multi-staining for macrophage marker F4/80 (Green) and myofibroblast marker α-SMA (purple) in CCl_4_-induced liver fibrosis model. **B** Quantitative data for the expression of α-SMA in CCl_4_-induced liver fibrosis model. **C** Quantitative data for the expression of F4/80 in CCl_4_-induced liver fibrosis model. **D** Quantitative data for the co-expression of F4/80 and α-SMA in CCl_4_-induced liver fibrosis model. All the data were shown as the mean ± s.d. **P < 0.01 and ***P < 0.001 vs. 0 weeks group. Scale bar, 20 µm

To further investigate the role of macrophage–myofibroblast transition (MMT) in liver fibrosis, Carbon tetrachloride (CCl4)-induced liver fibrosis animal model was employed. Immunofluorescent multi-staining was demonstrated to distinguish the co-expression of macrophage (F4/80) and myofibroblast (α-SMA) markers to identify MMT cells. The expression of F4/80 and α-SMA was both increased in the CCl4-treated mouse liver tissues (Fig. 2A–C). Furthermore, we also found that substantial numbers of co-expression of F4/80 and α-SMA cells in CCl4-treated mouse liver tissues, which account for almost half of the myofibroblast cells (Fig. 2D). The results suggested the MMT cells accounted for a significant proportion of myofibroblasts in active liver fibrosis.

### MMT in the NAFLD and AFLD model

**Fig. 3:**
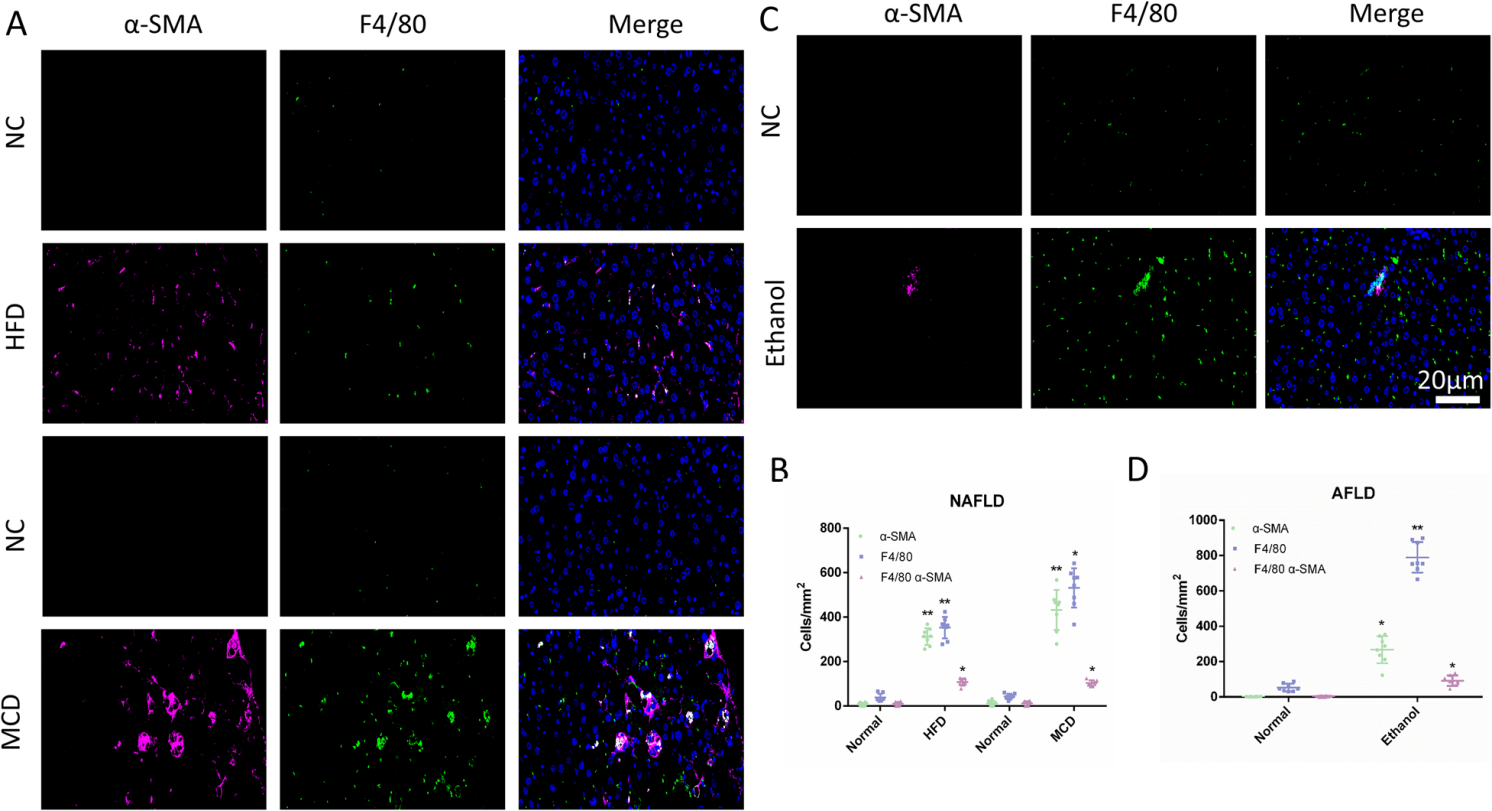
MMT in the NAFLD and AFLD model. **A** Representative images of immunofluorescent multi-staining for macrophage marker F4/80 (Green) and myofibroblast marker α-SMA (purple) in NAFLD model. **B** Quantitative data for the expression of F4/80 and α-SMA, and co-expression of F4/80 and α-SMA in NAFLD model. **C** Representative images of immunofluorescent multi-staining for macrophage marker F4/80 (Green) and myofibroblast marker α-SMA (purple) in AFLD model. **D** Quantitative data for the expression of F4/80 and α-SMA, and co-expression of F4/80 and α-SMA in AFLD model. All the data were shown as the mean ± s.d. *P < 0.05 and **P < 0.01vs. Normal group. Scale bar, 20 µm

Liver fibrosis is the result of liver damage repair caused by various injury [1]. Next, we explored the role of MMT in chronic liver injury. The model of nonalcoholic fatty liver disease (NAFLD) was established by the high-fat diet (HFD) and methionine- and choline-deficient diet (MCD). Compared to the respective control, the increased positive expression of macrophage (F4/80) and myofibroblast (α-SMA) was demonstrated in NAFLD model. Meanwhile, the double-labeled markers results indicated the cells of positive for F4/80 and α-SMA appeared compared with control tissues (Fig. 3A, B). Moreover, similar results were obtained in the alcoholic fatty liver disease (AFLD) model (Fig. 3C, D). Hence, MMT process was participated in the chronic liver injury.

### MMT cells have a predominant M2 phenotype

**Fig. 4:**
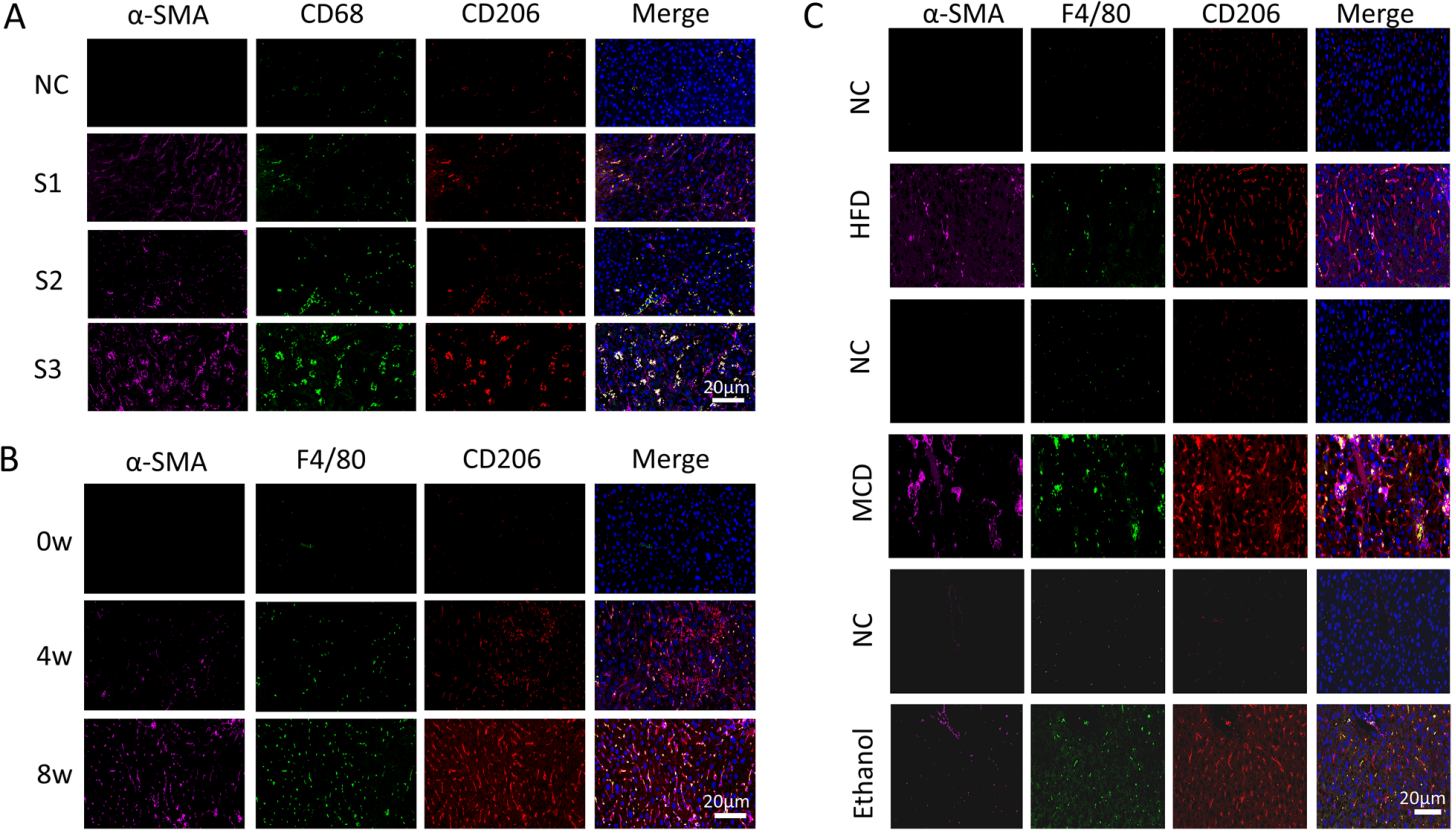
MMT cells have a predominant M2 phenotype. **A** Representative images identified M2-type cells (arrows) by triple-staining CD68 (green) CD206^+^ (red) α-SMA^+^(purple) in human liver fibrosis samples. **B** Representative images identified M2-type cells (arrows) by triple-staining F4/80 (green) CD206^+^ (red) α-SMA^+^(purple) in CCl4-induced liver fibrosis model. **C** Representative images identified M2-type cells (arrows) by triple-staining CD68 (green) CD206^+^ (red) α-SMA^+^(purple) in NFALD and AFLD model. All the data were shown as the mean ± s.d. *P < 0.05, **P<0.01. Scale bar, 20 µm

Previous studies have identified that MMT cells in kidney fibrosis are largely derived from M2 macrophages [8, 13]. We further proceeded to examine the M2 marker of CD206 in the clinical patient samples and animal models. As shown in Fig. 4A, CD68 and CD206 markers predominantly gathered in liver fibrosis samples. In addition, the most of F4/80 macrophages in animal model liver tissues were accompanied by co-expressed the CD206 marker (Fig. 4B, C). These results demonstrated that MMT cells had a predominant M2 phenotype in liver fibrosis and chronic liver injury.

## Discussion

Liver fibrosis is a condition that could be caused by many chronic liver injuries, such as viral hepatitis, excessive alcohol consumption and non-alcoholic steatohepatitis (NASH) [2]. Extracellular matrix (ECM) deposition is an important hallmark of liver fibrosis, and activated myofibroblasts plays a key role in ECM accumulation [14]. Recently, studies have identified that macrophages can act as a source of myofibroblasts directly through a process of macrophage-to-myofibroblast transition (MMT) [8, 15]. Our study indicated that MMT participated in the progression of liver fibrosis, which mainly occurred in macrophages with M2 phenotype.

Myofibroblasts, which are not detectable in healthy liver, are activated when the liver is damaged [4]. As myofibroblasts are the main source of ECM in fibrotic liver, and they are usually used as the main target of anti-fibrotic therapy. The initiation of myofibroblasts has been studied extensively and some have been identified, including HSCs, portal fibroblasts, liver-resident cells, bone marrow-derived cells like the mesenchymal stem cells (MSCs) and fibrocytes [16, 17]. Moreover, other sources of liver myofibroblasts have also been reported, such as epithelia cells through epithelial-to-mesenchymal transition (EMT) [6], endothelia cells through endothelial-to mesenchymal transition (EndoMT) [7]. Recently, studies have confirmed that macrophages can transform into myofibroblasts in the process of renal and lung fibrosis [11, 15]. Consistent with these researches, we also revealed that myofibroblasts derived from macrophages in liver fibrosis.

Macrophages are key players of human innate immunity, which mediate crucial immunomodulatory responses upon macrophage activation [12]. It has been well recognized that macrophages, especially activated macrophages, are associated with development of liver fibrosis indirectly by secreting cytokines and chemokines [18]. Under stimulation, macrophages can polarize into a pro-inflammatory M1 phenotype or anti-inflammatory M2 phenotype. M2 macrophages could secrete transforming growth factor-β (TGF-β) to promote myofibroblast proliferation and express pro-collagen I that contributes to fibrosis [19, 20]. TGF-β1, which is mainly secreted by activated macrophages, not only can promote the macrophages transiting from M1 into M2 phenotype, but also can induce MMT in renal fibrosis [15]. In our study, we found that MMT plays a significant role in the pathogenesis of liver fibrosis, with a predominant M2 phenotype.

## Conclusions

Consequently, MMT plays a crucial part in the progression of liver fibrosis. However, future studies are needed to further elucidate the mechanism of MMT process, thereby possibly extending therapeutic targets for preventing liver fibrosis.

## Data Availability

Not applicable.

## Abbreviations

AFLD: Alcoholic fatty liver diseases
CCL4: Carbon tetrachloride
ECM: Extracellular matrix
EndoMT: Endothelial-to mesenchymal transition
EMT: Epithelial-to-mesenchymal transition
HFD: High-fat diet
HSCs: Hepatic stellate cells
MCD: Methionine- and choline-deficient diet
MFs: Myofibroblasts
MMT: Macrophages into myofibroblasts
MSCs: Mesenchymal stem cells
NAFLD: Nonalcoholic fatty liver disease
NASH: Non-alcoholic steatohepatitis
TGF-β: Growth factor-β
α-SMA: α-smooth muscle actin
